# Chronic Rhinosinusitis: MALDI-TOF Mass Spectrometry Microbiological Diagnosis and Electron Microscopy Analysis; Experience of the 2nd Otorhinolaryngology Clinic of Cluj-Napoca, Romania

**DOI:** 10.3390/jcm9123973

**Published:** 2020-12-08

**Authors:** Ionuț Isaia Jeican, Lucian Barbu Tudoran, Adrian Florea, Mirela Flonta, Veronica Trombitas, Anda Apostol, Mihai Dumitru, Maria Aluaș, Lia Monica Junie, Silviu Albu

**Affiliations:** 1 Department of Head and Neck Surgery and Otorhinolaryngology, University Clinical Hospital of Railway Company, Iuliu Hatieganu University of Medicine and Pharmacy, 16-20 Republicii, 400015 Cluj-Napoca, Romania; jeican.ionut@umfcluj.ro (I.I.J.); veronicatrombitas@gmail.com (V.T.); andapstl@yahoo.com (A.A); silviualbu63@gmail.com (S.A.); 2 Department of Anatomy and Embryology, Iuliu Hatieganu University of Medicine and Pharmacy, 3-5 Clinicilor, 400006 Cluj-Napoca, Romania; 3 Electron Microscopy Laboratory Prof. C. Craciun, Faculty of Biology and Geology, Babes-Bolyai University, 5-7 Clinicilor, 400006 Cluj-Napoca, Romania; lucian.barbu@ubbcluj.ro; 4 Electron Microscopy Integrated Laboratory, National Institute for R&D of Isotopic and Molecular Technologies, 67-103 Donath, 400293 Cluj-Napoca, Romania; 5 Department of Cell and Molecular Biology, Iuliu Hatieganu University of Medicine and Pharmacy, 6 Louis Pasteur, 400349 Cluj-Napoca, Romania; aflorea@umfcluj.ro; 6 Infectious Diseases Hospital of Cluj-Napoca, 23 Iuliu Moldovan, 400348 Cluj-Napoca, Romania; m.flonta@yahoo.com; 7 Department of Head and Neck Surgery and Otorhinolaryngology, Bucharest University Emergency Hospital, 169 Splaiul Independenței, 010271 Bucharest, Romania; orldumitrumihai@yahoo.com; 8 Department of Anatomy and Embryology, Carol Davila University of Medicine and Pharmacy, 1 Frosa Sarandy, 050474 Bucharest, Romania; 9 Department of Oral Health, Iuliu Hatieganu University of Medicine and Pharmacy, 15 Victor Babes, 400012 Cluj-Napoca, Romania; 10 Department of Microbiology, Iuliu Hatieganu University of Medicine and Pharmacy, 6 Louis Pasteur Street, 400349 Cluj-Napoca, Romania; mjunie@umfcluj.ro

**Keywords:** chronic rhinosinusitis (CRS), odontogenic sinusitis, biofilms, MALDI-TOF MS, endoscopic sinus surgery

## Abstract

(1) Background: Chronic rhinosinusitis (CRS) represents a wide range of infectious-inflammatory processes affecting, simultaneously, the nose and paranasal sinuses mucosa. The paper presents outcomes of the investigation of CRS microbiological characteristics in a group of 32 patients. (2) Methods: The purulent samples were collected during functional endoscopic sinus surgery. Agar plates were incubated and examined. All types of colonies were identified using Matrix-Assisted Laser Desorption - Ionisation-Time of Flight Mass Spectrometry (MALDI-TOF MS). For scanning electron microscopy, samples were fixed and sputter-coated with 10 nm gold and analyzed using a scanning electron microscope. For transmission electron microscopy, samples were fixed, postfixed, and dehydrated. After polymerization, ultrathin sections were collected on carbon coated copper grids and analyzed with Jeol JEM1010 TEM. (3) Results: Positive microbiological diagnosis was obtained in 62.5% of cases. The most frequent species found are *Staphylococcus aureus* and *Streptococcus constellatus* subsp. *pharyngis*. Corynebacterium aurimucosum and Eggerthia catenaformis were unreported species in CRS until the present. Biofilm was evidenced in 43.7% of sinus mucosa samples. Ciliary disorientation, atrophy, and no ciliated cells were also identified. (4) Conclusion: The microbial factor—pathogen or opportunistic—is one of the most important pathological links in chronic rhinosinusitis. MALDI-TOF MS allows easily and quickly identification of germs.

## 1. Introduction

Chronic rhinosinusitis (CRS) is defined as inflammation of the nose and paranasal sinuses [1]. CRS is considered one of the most widespread chronic diseases, representing a financial burden for health care systems [2]. An accurate diagnosis of CRS is difficult, CRS symptoms being common to other rhinologic conditions [3]. As such, the prevalence data may be unreliable [4].

European Position Paper on Rhinosinusitis and Nasal Polyps 2020 (EPOS) classification distributes CRS in primary and secondary, divided each into localized and diffuse [1]. In primary CRS, the disease is divided in type 2 or non-type 2 of inflammation (type 2 inflammation is characterized by cytokines IL-4, IL-5, and IL-13, activation and recruitment of eosinophils and mast cells). Secondary CRS can also be localized (odontogenic, fungal ball, tumor) or diffuse. Primary CRS type 2 include CRS with nasal polyps (CRSwNP)—approximately 25–30% of cases [5].

If two decades ago CRS research was based on an infection model of disease, nowadays it has largely given way to an inflammation-based model. At present, it is known that both injured and healthy sinuses are populated by microbes, including potentially pathogen species [6]. The microbiome is the collective genome of the microorganisms which populate the ecosystem of the paranasal sinus. CRS pathogenesis involves a general process of imbalance, or dysbiosis, in its bacterial community structures [7,8].

Matrix-assisted laser desorption/ionization time-of-flight mass spectrometry (MALDI-TOF MS) has become a gold standard for microbial identification in clinical laboratories [9]. MALDI-TOF MS is rapid, reliable, and a high-throughput diagnostic tool for the identification of microorganisms [10]. MALDI-TOF MS diagnosis correlated with high performance microbiological techniques and molecular genetics analyses is at the forefront of research in CRS.

The purpose of our study was investigation of microbiological characteristics (the microbial etiological spectrum, the presence of the biofilm), through MALDI-TOF MS and electron microscopy analysis, of CRS in northwestern Romania.

## 2. Experimental Section

### 2.1. Study Design and Population

In our approach, we adopted an observational study of patients with CRS admitted in the 2nd Otorhinolaryngology Clinic, University Clinical Hospital of Railway Company, Cluj-Napoca, between February 2018–December 2019.

The study included 32 patients diagnosed with CRS according to the EPOS criteria: minimum two symptoms (nasal blockage/obstruction/congestion or nasal discharge, ±facial pain/pressure, ±reduction of smell), and either endoscopic signs (nasal polyps, mucopurulent discharge from middle meatus, edema/mucosal obstruction in middle meatus) and/or CT changes (mucosal changes within the ostiomeatal complex and/or sinuses) [1].

We excluded from the study patients under the age of 18 years, or those with previous rhinosinusal surgery, under antibiotic/topic corticosteroid treatment less than three weeks before operation, malignant tumors/associated autoimmune diseases, pregnant women, patients with low ciliary function (cystic fibrosis, Kartagener syndrome), and patients with insufficient collected histological sample (sinus mucosa).

The written Informed Consent of patients was obtained a day before the surgery. Patients agreed with collection of purulent and mucosal samples and with the sample investigation through MALDI-TOF MS and electron microscopy. The access to patient’s files, personal data such as samples were allowed only to the research team, respecting confidentiality and privacy of participants.

The study protocol was approved by Iuliu Hatieganu University of Medicine and Pharmacy Ethics Committee under the No. 87/01.02.2018.

### 2.2. Sampling

After induction of general anesthesia by oro-tracheal intubation, the nasal vestibule was cleaned with iodine, the nasal cavity by saline solution, and a local vasoconstricting agent was instilled. The purulent samples for microbiological examination were collected with two swabs from the affected sinus, during Functional Endoscopic Sinus Surgery (FESS). The samples were transported to the microbiology laboratory in Amies medium and brain heart infusion broth with thioglycolate. Three mucosal biopsies were obtained from each patient from the same side, during FESS: one for histopathology and two for scanning electron microscopy.

### 2.3. Microbiological Diagnostic Procedures

Within one hour from collecting, the purulent samples were taken to the laboratory and culture was rapidly initiated so that the maximum time between collection and culture was 2 h. Samples placed on Amies medium were cultured on Columbia Blood Agar (Oxoid, Basingstoke, Hampshire, United Kingdom), Chocolate Agar (Oxoid, Basingstoke, Hampshire, United Kingdom), MacConkey Agar (Oxoid, Basingstoke, Hampshire, United Kingdom) and were incubated at 37 °C in an atmosphere containing 5% CO_2_ for 24 h. Samples collected on broth were cultured on Schaedler Agar with Sheep Blood (Oxoid, Basingstoke, Hampshire, United Kingdom) and media were placed in an anaerobic atmosphere in a jar at 37 °C for 48 h.

Plates were examined at 24 and 48 h for growth. A presumptive identification was performed on colony color, shape, hemolysis, and Gram stain morphology. Finally, all types of colonies were identified using MALDI-TOF mass spectrometer Biotyper Bruker, Bremen, Germany. Susceptibility of the isolates was determined by the disk diffusion method (Kirby–Bauer) on Mueller–Hinton Agar with discs from Oxoid, Basingstoke, Hampshire, United Kingdom and according to European Committee on Antimicrobial Susceptibility Testing (EUCAST) standard.

### 2.4. Scanning Electron Microscopy (SEM)

Samples were fixed in glutaraldehyde 2.7% for 2 h, washed with PBS and then with distilled water, then left to dry. Dried samples were glued to a support with silver paste and the material was further sputter-coated with 10 nm gold. Prepared samples were analyzed using a Hitachi SU8230 Tokio, Japan scanning electron microscope at 30 kV. All samples were compared to a control (unaffected) tissue image. The aspect of the mucosa, biofilm identity, and the ciliary patterns were investigated. For cultured bacteria, samples were prepared by fingerprint-method, and fixed for 15 min in osmium tetroxide vapors. Samples were sputter-coated with 10 nm gold in an Agar Automatic Sputter Coater (United Kingdom).

### 2.5. Transmission Electron Microscopy (TEM)

The glutaraldehyde (2.7% in 0.1M PBS) 75 min fixed tissues were rinsed 3 times with 0.15M PBS, pH 7 for 1 h each, and postfixed in 2% osmium tetroxide for 1 h. Dehydration was accomplished in a series of mixtures of water with component A (a water-soluble aliphatic polyepoxide) of the Durcupan ACM water-soluble embedding medium (Fluka, Munich, Germany) as follows: 50% component A with 50% water for 30 min; 70% component A with 30% water for 45 min; 90% component A with 10% water for 45 min; 2 × 100% component A for 90 min. The dehydrated tissue was then placed in a polymerization mixture of the components A through D (components B and C are hardeners; component D is the plasticizer) according to the manufacturer’s protocol and left overnight in a refrigerator for final mixing and embedding. Polymerization was performed in a freshly prepared mixture of the above composition for 4 days at 42 °C. Ultrathin sections, about 50 nm thick, were obtained using a Leica Ultracut ultramicrotome and a diamond knife (Leica Microsystems, Bensheim, Germany). The sections were collected on copper grids covered by a thin layer of colodium and carbon. Final staining of the sections included treatment with uranylacetate for 15 min and with lead citrate for 9 min. Microscopic examinations were carried out with a Jeol Electron Microscope 1010 transmission electron microscope (Tokio, Japan).

## 3. Results

Odontogenic CRS was most common (40.6%, *n* = 13/32), followed by CRSwNP (34.4%, *n* = 11/32), CRSsNP (15.6%, *n* = 5/32), and sphenoid CRS (9.4%, *n* = 3/32). Demographic and diagnostic data of the 32 patients included in the study are presented in Table 1.

Controlled essential hypertension was found in 37.5% (*n* = 12/32) of patients, and type 2 diabetes mellitus in 12.5% (*n* = 4/32) of patients. Aspirin exacerbated respiratory disease (Widal syndrome) was presented in 36.3% (*n* = 4/11) of patients with CRSwNP. Two patients were admitted for revision endoscopic surgery.

### 3.1. Microbiological Diagnosis

Positive microbiological diagnosis from purulent samples was obtained in 62.5% of all cases (*n* = 20/32) (Table 1): CRSsNP/CRSwNP/odontogenic 40%/81.8%/69.2% (*n* = 2/9/9) of the cases of the respective subgroup.

In 28% (*n* = 9/32) of purulent samples, bacterial associations were evidenced (polymicrobial CRS), mostly from patients with odontogenic CRS.

Seventeen bacterial species were isolated, of which 9 were Gram-positive (52.9%) and 8 Gram-negative (47.1%). The most frequent species found were *Staphylococcus aureus* and *Streptococcus constellatus* subsp. *pharyngis*.

### 3.2. Electron Microscopy Analysis

**Figure 1:**
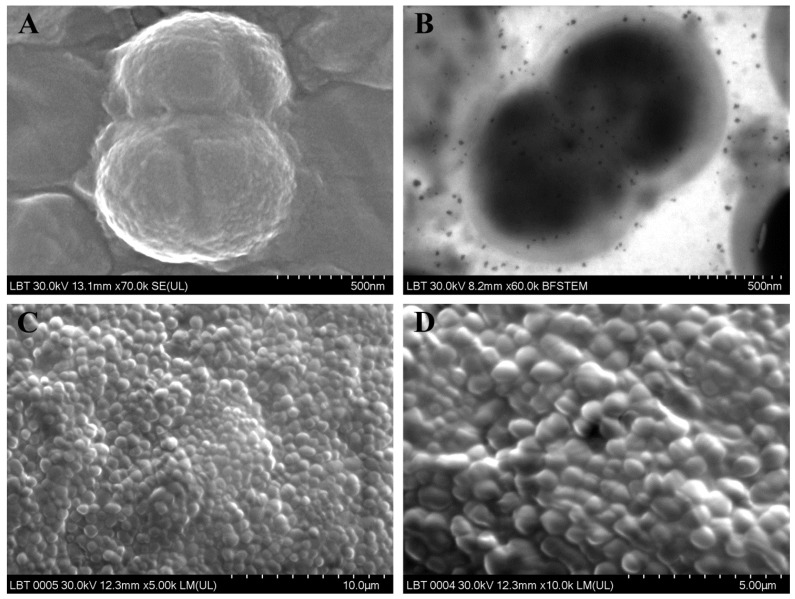
Ultrastructural features of the bacteria identified in patients with CRS: (**A**,**B**) *Enterococcus faecalis*; (**C**,**D**) *Parvimonas micra* (**A**,**C**,**D**: SEM images; **B**: TEM image).

**Figure 2:**
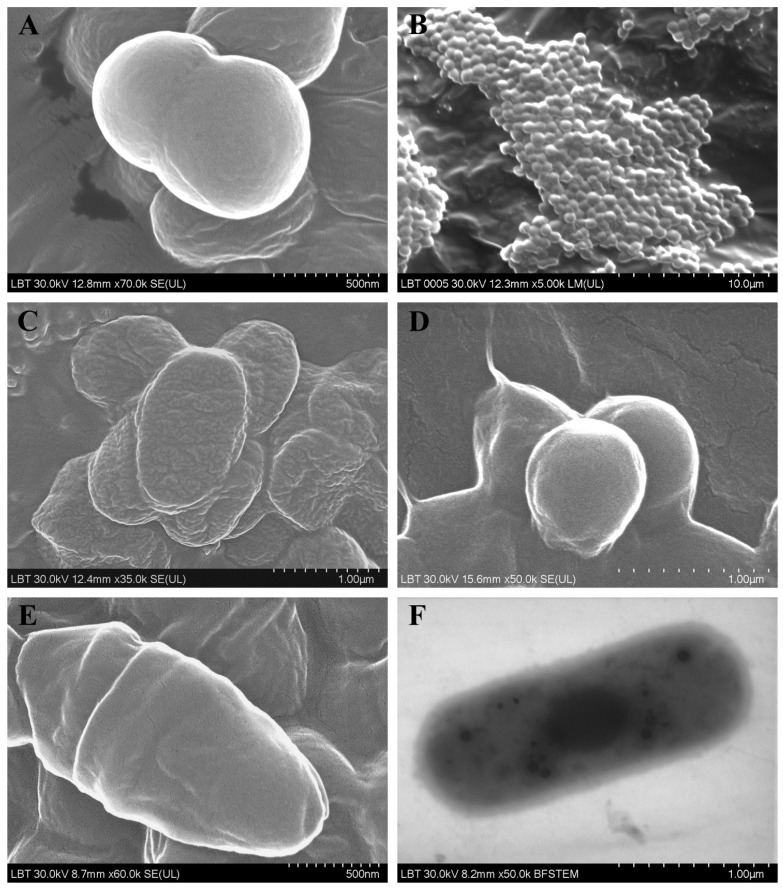
Ultrastructural features of the bacteria identified in patients with CRS: (**A**) Staphylococcus aureus; (**B**) *Streptococcus constellatus* subsp. *pharyngis*; (**C**) *Klebsiella pneumoniae*; (**D**) *Finegoldia magna*; (**E**) *Corynebacterium aurimucosum*; (**F**) *Eggerthia catenaformis*. (**A**–**E**: SEM images; **F**: TEM image).

Figure 1 and Figure 2 include the most relevant images of the ultrastructural features of the isolated species.

**Figure 3:**
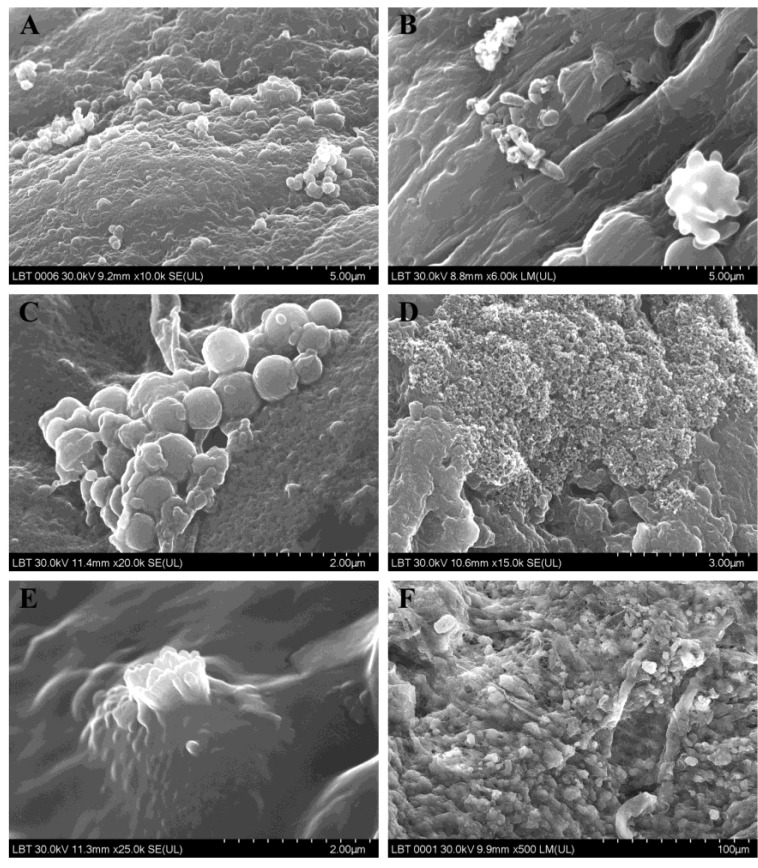
Microbial biofilms in patients with CRS: (**A**–**C**) adherence of microbial elements to the surface of the sinus mucosa; (**D**) biofilm of *Staphylococcus aureus*; (**E**) biofilm of *Klebsiella pneumoniae*; (**F**) mature biofilm (SEM images).

Biofilm was evidenced in 43.7% (n = 14/32) of sinus mucosa samples (Figure 3), 15.6% (n = 5/32) of which were from patients having a negative microbiological test.

**Figure 4:**
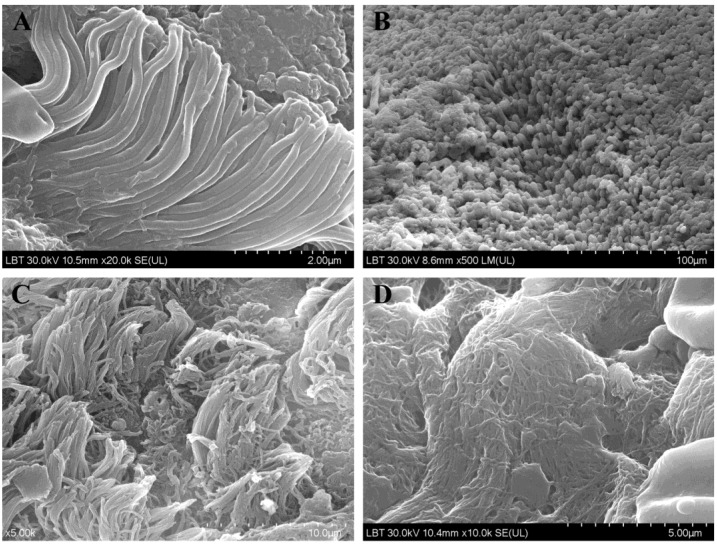
Aspects of sinus mucosa ultrastructure in CRS patients (SEM): cilia with normal aspect (**A**), area dense in microvilli (**B**), ciliary disorientation (**C**), and ciliary atrophy (**D**).

On 84% (*n* = 27/32) of the sinus mucosa samples ciliary disorientation was found (Figure 4C), while atrophy (Figure 4D) was found in 37.5% (*n* = 12/32). There were no ciliated cells left in 15.6% (*n* = 5/32) of the samples. We could not correlate these modifications of the cilia with the result of the microbiological examination.

## 4. Discussion

Microbiome analyses of sinusal samples with sequencing techniques showed that less diversity in the microbial community rather than an increased overall bacterial load seems to characterize CRS compared to the healthy state [11]. Bacterial diversity was reduced in CRSsNP compared to that in the controls but not in CRSwNP, emphasizing the difference in pathophysiology between these two phenotypes [12].

Microbiome analyses raise an important issue: they question the significance of the results of conventional lab culturing in general. A culture of the sinus obtained via the nose will always grow microbes because the nose is not sterile and causality in CRS is not established by a positive culture [1]. The results of the microbiological tests do not differ whether the sample is harvested on swabs from the opened affected sinus during FESS or taken on swabs preoperatively from the middle meatus [13].

It seems that the development of certain culture species depends rather on their ability to grow on culture media than on their real abundance or pathogenetic significance [13,14]. Conventional lab culture tends to select abundant aerobic microorganisms, growing rapidly, such as *Staphylococcus aureus* [14], reported by the majority of studies to be the most frequent etiological agent isolated in CRS [15,16]. While identification rates of *Staphylococcus aureus* are comparable between health and CRS patients, alterations in the virulence and activity of *Staphylococcus aureus* could be a possible etiological or exacerbating factor in the CRS patients [7]. Using this model, we may assume that virulence modifications of human-associated microbes determines the onset of CRS with opportunistic agents, more and more frequently encountered nowadays.

The limit of our study is the low number of patients. Consequently, we cannot indicate the study power. The fact that in 37.5% of patients we did not obtain a positive microbiological examination may be due to the multiple antibiotic treatments previously tried by FESS—during acute episodes (but the last antibiotic treatment was not made with less than three weeks before surgery), which influenced the result or due to the organization of the mature biofilm.

The study shows the clinician the significant weight of opportunistic germs in CRS, as well as etiological spectrum differences between CRS types. It also identifies bacteria species unreported previously. Using MALDI-TOF MS in microbiological diagnosis, studies conducted on significant samples could complete our results.

Bacteria exist in two distinct forms: biofilm and planktonic. Biofilm is the preferred state in which an estimated 99% of bacteria exist [17]. If, in the beginning, studies identified biofilms on the sinus mucosa surface of most CRS patients, subsequent studies sometimes evidenced an absence of biofilms in patients with the disease and their presence on the sinus mucosa of healthy individuals. This variation of results revealed the complex, multifactorial pathophysiology of CRS, and the apparently contradictory conclusions were attributed to the different sensitivity and specificity of the techniques of biofilm analysis [18,19]. Moreover, the subjectivity of the observer may also be a source of error, while the variations of the methods of preparation of sinus mucosa fragments may lead to the loss or diminution of the biofilm.

Odontogenic CRS is distinct from other types of CRS because it originates from odontogenic infection (maxillary molar teeth, maxillary dental trauma) or dental procedures (extraction or implants) [20]. Patients have nonspecific sinusal symptoms and minimal dental complaints. Dentists or radiologists can easily miss this diagnosis and otolaryngologists are often responsible for recognizing odontogenic CRS [21].

According to other studies [22,23], we identified anaerobic bacteria and polymicrobial growth in odontogenic CRS. Bacteria associated with odontogenic CRS are consistent with oral flora, present in subgingival plaques [21]. In comparison with rhinogenic CRS, we frequently found *Streptococcus constellatus* in odontogenic CRS.

*Streptococcus constellatus* subsp. *pharyngis*, a component species of *Streptococcus anginosus* group [24], is a commensal germ of the oral cavity and pharynx; it can become pathogenic and lead to an infection of the surrounding or distant sites after mucosal disruption [25]. It has been evidenced in complicated rhinosinusites [26]. We isolated it both as a unique germ and associated with *Staphylococcus lugdunensis*, *Klebsiella pneumoniae*, and *Parvimonas micra (*Table 1. The association between *Streptococcus* *constellatus* subsp. *pharyngis* and *Staphylococcus lugdunensis* has already been reported [27].

In immunologically competent patients with CRS, *Pseudomonas aeruginosa* is usually evidenced in association with other species (polymicrobial CRS) [28,29]. In our study, we isolated *Pseudomonas aeruginosa* associated with *Staphylococcus aureus* in a 76-year-old female patient with odontogenic CRS, multiple cardiovascular co-morbidities, and type 2 diabetes mellitus.

*Dialister pneumosintes* is a known endodontic and periodontal anaerobic pathogen found in necrotic pulp, subgingival plaque, and deep periodontal pockets [30]. We isolated it in a 40-year-old immuno-competent female patient with odontogenic CRS. *Dialister pneumosintes* has already been reported in chronic maxillary sinusitis of odontogenic origin [30,31]. 

The results of the microbiological examinations performed in our study evidenced a number of opportunistic germs. *Enterobacter aerogenes* is an opportunistic bacterium, involved in several nosocomial infectious foci in Europe in the past [32]. We isolated it in odontogenic CRS. Other studies have also reported it in CRS [33,34]. *Finegoldia magna* (formerly *Peptostreptococcus magnus*), anaerobe, is part of the normal human mucocutaneous flora, isolated from deep organ abscesses, obstetric and gynecological sepsis, intraoral infections, and sinusitis [35,36]. In addition, *Prevotella* is an opportunistic anaerobic rod, involved in chronic periodontal infections [37] and CRS [38]. We isolated these species both from CRSwNP and odontogenic CRS patients.

*Bacillus subtilis* is an aerobic, endospore-forming, opportunistic pathogen, common soil inhabitant. It may frequently contaminate foods and is widely spread in hospital environments [39]. We isolated it in association with *Staphylococcus aureus* in a male patient of low socio-economic status, lacking corporal hygiene. Like *Enterococcus faecalis*, *Bacillus subtilis* may be located intranasally by nose picking with a dirty hand.

*Staphylococcus lugdunensis* is a commensal bacterium that colonizes the skin [40,41]. Nasal colonization may explain the presence of *Staphylococcus lugdunensis* in head and neck infections. Phenotypic identification of *Staphylococcus lugdunensis* is challenging, but the implementation of MALDI-TOF MS has provided laboratories with a fast and cost-effective identification tool [42]. It has also been reported in CRS [43].

*Corynebacterium aurimucosum* was isolated from healthy female urogenital tract samples and is considered part of the normal microbial community [44]. We have not found any reporting in CRS patients. In our study, *Corynebacterium aurimucosum* was evidenced in association with *Eggerthia catenaformis* in a 39-year-old immuno-competent female patient with odontogenic CRS. *Eggerthia catenaformis* was isolated from human stools and rarely reported in human diseases [45]. *Eggerthia catenaformis* has been reported in bacterial infections related to dental abscesses [46,47].

*Parvimonas micra* is belongs to the normal commensal flora of the gastrointestinal tract and was frequently isolated from lesions of apical periodontitis [48,49].

Chronic inflammation also determines changes in cyto-architecture, detectable by electron microscopy: a loss of ciliated cells and an increasing number of nonciliated columnar cells with microvilli, increased number of microvilli, ciliary disorientation, no ciliated cells, short cilia indicating ciliogenesis and regeneration of epithelium, mucus abundant [50,51]. The combination of SEM and TEM techniques increases the accuracy of results.

## 5. Conclusions

This paper highlights that pathophysiological mechanisms in CRS are varied but not mutually exclusive; on the contrary, they are closely intricate. Most probably, the reality behind the mechanisms known until now to be involved in the onset and maintenance of CRS are much more complex. The certainty for the clinician is that the microbial factor—pathogenic or opportunistic—will remain one of the most important pathological links in chronic rhinosinusitis.

MALDI-TOF MS allowed us to identify unreported species until the presence in CRS, as well as identification of a number of opportunistic germs. Future studies are needed for research the virulence modifications of human-associated microbes in CRS with opportunistic agents.

## Figures and Tables

**Table 1:** Distribution of the CRS patient group and bacterial species isolated.

Gender Distribution				
Gender	Male	Female		
No. of Patients	11	21		
Age Distribution				
No. of Patients	CRSsNP (Primary CRS Diffuse Non-Type 2)	CRSwNP (Primary CRS Diffuse Type 2)	Odontogenic CRS (Secondary CRS Unilateral)	Sphenoid CRS
32	5	11	13	3
Bacterial Species Isolated				
	Species identified	Gram Staining Tinctorial Affinity	Metabolism	Biofilm
CRSsNP (Primary CRS diffuses non-type 2)	*Enterococcus faecalis* (Figure 1A,B)	G+	Facultatively anaerobic	Absent
	sensitive to ampicillin, ciprofloxacin, vancomycin			
	*Pseudomonas aeruginosa*	G−	Aerobic	Present
	*Staphylococcus aureus* (Figure 2A)	G+	Aerobic	
	*Pseudomonas*: resistant to sulfamethoxazole-trimethoprim, amoxicillin-clavulanate, cefuroxime; sensitive to colistin, ciprofloxacin, ceftazidime. *Staphylococcus*: methicillin-sensitive.			
CRSwNP (Primary CRS diffuse type 2)	*Staphylococcus aureus*	G+	Aerobic	Present
	*Bacillus subtilis*	G+	Aerobic	
	*Staphylococcus*: resistant to oxacilin, clindamycin; sensitive to: ciprofloxacin, gentamicin, sulfamethoxazole-trimethoprim			
	*Staphylococcus lugdunensis*	G+	Aerobic	Absent
	sensitive to: oxacillin, ciprofloxacin, gentamicin, sulfamethoxazole-trimethoprim			
	*Klebsiella pneumoniae* (Figure 2C)	G−	Facultatively anaerobic	Present
	resistant to ampicillin; sensitive to: amoxicillin-clavulanate, ciprofloxacin, gentamicin, sulfamethoxazole-trimethoprim			
	*Staphylococcus aureus* (2 samples) *	G+	Aerobic	Present in 1 of 2 samples
	methicillin-sensitive			
	*Finegoldia magna* (Figure 2D)	G+	Anaerobic	Absent
	*Prevotella oris*	G−	Anaerobic	Present
	*Prevotella buccae*	G−	Anaerobic	
	*Staphylococcus lugdunensis*	G+	Anaerobic	
	*Staphylococcus*: methicillin-sensitive			
	*Haemophilus influenzae*	G−	Facultatively anaerobic	Absent
	sensitive to: ampicillin, amoxicillin-clavulanate, sulfamethoxazole-trimethoprim			
	*Streptococcus constellatus* subsp. *pharyngis*	G+	Anaerobic	Absent
	*Bacteroides pyogenes*	G−	Anaerobic	
	*Streptococcus*: sensitive to clindamycin, erythromycin			
Odontogenic CRS (Secondary CRS unilateral)	*Enterobacter aerogenes*	G−	Facultatively anaerobic	Present
	*Finegoldia magna*	G+	Anaerobic	
	*Enterobacter*: resistant to ampicillin, amoxicillin-clavulanate; sensitive to: cephalosporins, gentamicin, sulfamethoxazole-trimethoprim			
	*Dialister pneumosintes*	G−	Anaerobic	Absent
	*Streptococcus constellatus* subsp. *pharyngis (2 samples) (*Figure 2B)	G+	Anaerobic	Absent
	sensitive to clindamycin, erythromycin			
	*Staphylococcus lugdunensis*	G+	Aerobic	Absent
	*Prevotella buccae*	G−	Anaerobic	
	*Staphylococcus*: sensitive to: ciprofloxacin, gentamicin, sulfamethoxazole-trimethoprim			
	*Staphylococcus aureus*	G+	Aerobic	Present
	methicillin-sensitive			
	*Klebsiella pneumoniae*	G−	Facultatively anaerobic	Present
	*Streptococcus constellatus* subsp. *pharyngis*	G+	Facultatively anaerobic	
	*Parvimonas micra* (Figure 1C,D)	G+	Anaerobic	
	*Streptococcus*: sensitive to clindamycin, erythromycin			
	*Staphylococcus lugdunensis*	G+	Aerobic	Present
	*Streptococcus constellatus* subsp. *pharyngis* *	G+	Anaerobic	
	*Staphylococcus*: resistant to oxacillin, ciprofloxacin, gentamicin; sensitive to: sulfamethoxazole-trimethoprim, clindamycin			
	*Corynebacterium aurimucosum* (Figure 2E)	G+	Aerobic	Absent
	*Eggerthia catenaformis* (Figure 2F)	G+	Anaerobic	

* Patient with revision endoscopic surgery.
